# Precise construction of DNA origami‐based materials for functional regulation on biological interface

**DOI:** 10.1002/smo.20230032

**Published:** 2024-03-04

**Authors:** Yushuai Wu, Xiaohui Wu, Run Tian, Yiming Wang, Baoquan Ding, Qiao Jiang

**Affiliations:** ^1^ CAS Key Laboratory of Nanosystem and Hierarchical Fabrication CAS Center for Excellence in Nanoscience National Center for NanoScience and Technology Beijing China; ^2^ University of Chinese Academy of Sciences Beijing China; ^3^ School of Materials Science and Engineering Zhengzhou University Zhengzhou China

**Keywords:** bio‐interface, biological application, DNA origami, self‐assembly

## Abstract

Precise design and control of molecular self‐assembly as living creatures are exciting ideas in the field of nanotechnology. Characterized with predesigned geometries and accurate spatial addressability, programmable DNA origami nanostructures have been recognized as optimized tools for assembling multiple functional components. A variety of biomolecules can be attached to the nanoscale drawing boards in a site‐specific fashion, thus facilitating the precise construction of DNA origami‐based materials for studies on biological interface. In this minireview, we highlight the recent advances in the precise construction of DNA origami‐based materials with artificial bio‐structures and/or biomimicking functions. The regulation of biological functions by these DNA origami‐engineered assemblies at the bio‐interface has been summarized and discussed.

## Graphical abstract

With predesigned geometries, precise addressability, versatile programmability, and remarkable biocompatibility, DNA origami yields a variety of promising tools for self‐assembling multiple functional moieties and precise construction of materials with artificial bio‐structures and/or mimicking biofunctions. Recent progress in the growing field and future application are discussed in this minireview.

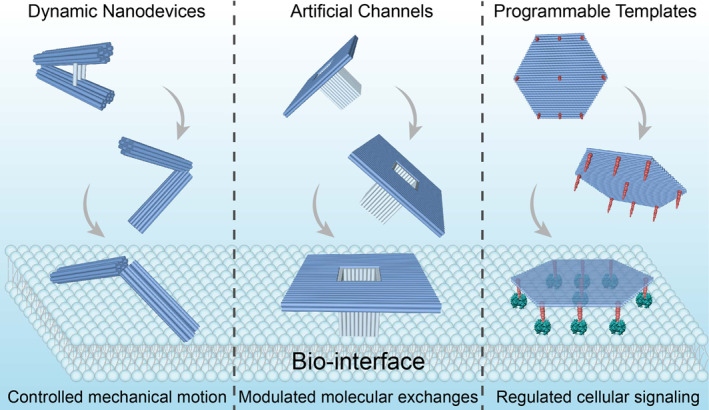

## 1 INTRODUCTION

Biological materials are a kind of synthetic material that mimic biological substances and natural materials in structures and functions. To date, a wide variety of methodologies have been introduced for the construction of synthetic materials with artificial bio‐structures and/or mimicking biofunctions.^[1]^ In contrast to conventional synthetic strategies,^[2]^ the concept of using DNA molecules, the genetic materials in almost all the living creatures, as building blocks to fabricate arbitrary structures and realize the pre‐designed functions were proposed by Nadrian Seeman in 1982.^[3]^ Significant progresses have been made in the field of structural DNA technology, including the creation of delicate architectures ranging from nanometer scale^[4]^ to micrometer scale,^[5]^ from static to dynamic, and from simple control to precise response to environmental signals for sophisticated functions.[^4^, ^6^] Since the first report on DNA origami techniques was published in 2006,^[7]^ various origami‐based nanomaterials with delicate structures, programmable functionalities and remarkable biocompatibility have attracted much research attention,^[8]^ especially for adapting them to study and regulate biological functions.

Predesigned origami structures with arbitrary geometries can serve as addressable canvases for assembling functional components. These bio‐inspired, sophisticated, origami‐based nanoarchitectures have been designed as nanodevices for controlled mechanical motions and precise sensing, as selective DNA‐based nanochannels for modulation of molecular exchanges, as well as nanoscale drawing boards for molecular patterns to regulate cellular signaling.

**FIGURE 1:**
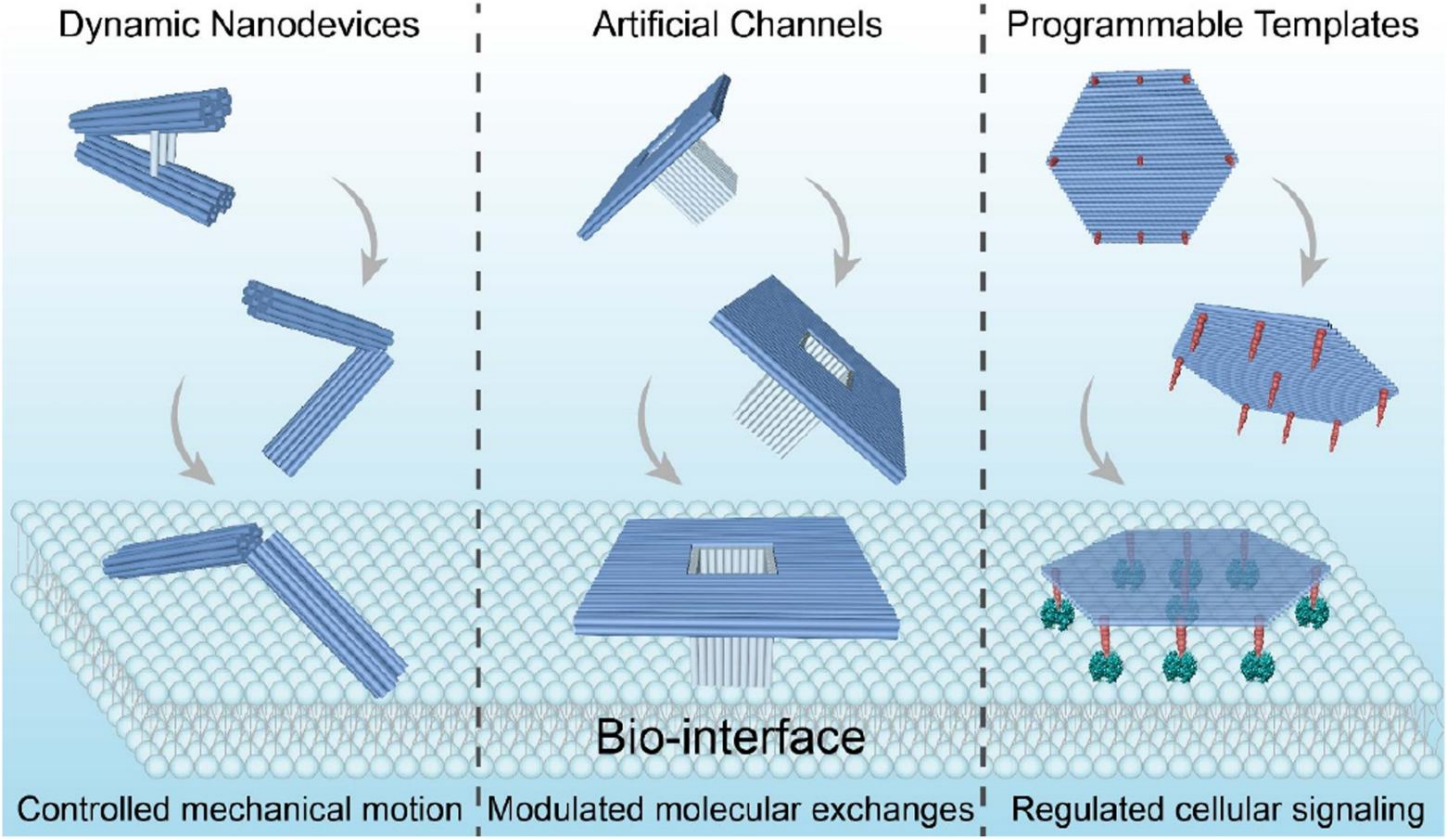
DNA origami‐based materials for functional regulation on bio‐interface.

DNA origami is typically assembled from a long single‐stranded DNA (ssDNA) scaffold and hundreds of short ssDNA helpers, folding into a pre‐designed structure with programmed and uniform geometry. With an entirely addressable surface, DNA origami structures can serve as guiding scaffolds for the precise arrangement of biological payloads, such as small molecular drugs,^[9]^ dyes,^[10]^ nanoparticles,^[11]^ proteins,^[12]^ peptides,^[13]^ nucleic acids,^[14]^ etc. These sophisticated and hybrid nanoarchitectures consisting of origami templates and functional cargos can usually be constructed by ssDNA modification of biomolecules and their accurate attachment to the DNA nanostructures via DNA hybridization. The advances of DNA origami‐based drug delivery platforms have been summarized elsewhere.^[15]^ The DNA origami‐based nanoassemblies with rationally precise types, numbers and patterns of desired functional moieties enable unique biological functions, which can be used as optimal tools to study and modulate biological signals and processes. Here, we will focus on the recent progresses in DNA origami‐based materials for bio‐interface studies, including the precise construction of origami nanodevices and nanoprobes for desired structural motions and biosensing, DNA nanopores and selective barriers for controlling molecular exchanges on artificial membranes and biological membranes, as well as the origami‐templated patterns of biomolecules for cell signaling studies (Figure 1).

## 2 CONSTRUCTION OF DNA ORIGAMI NANODEVICES FOR BIOLOGICAL STUDIES

In the past few decades, the precise design and construction of dynamic DNA origami‐based nanostructures have seen significant advancements.^[16]^ Much progress in precise construction of nanoprobes and nanodevices based on DNA technology has been reported, such as nanoscale probes for high‐performance bioimaging,^[17]^ membrane‐anchored DNA tetrahedrons for signaling pathway study,^[18]^ DNA structure‐based single‐molecule CRISPR machine for spatially resolved search,^[19]^ and multilayer DNA‐based programmable gate arrays for DNA computing.^[20]^ The following section highlights the recent advances in the precise assembly of DNA origami‐based nanoprobes that can detect and modulate specific biological behaviors, including origami tweezers or springs for mechanical force sensing, origami shells for viral detection and inhibition, origami containers for enzymatic studies, and origami vehicles for cellular entry. Several dynamic origami nanodevices that move in a biomimetic fashion, such as ratchet‐like rotary motors, mechanical arms, and tweezer‐like engines, have also been included in this section.

**FIGURE 2:**
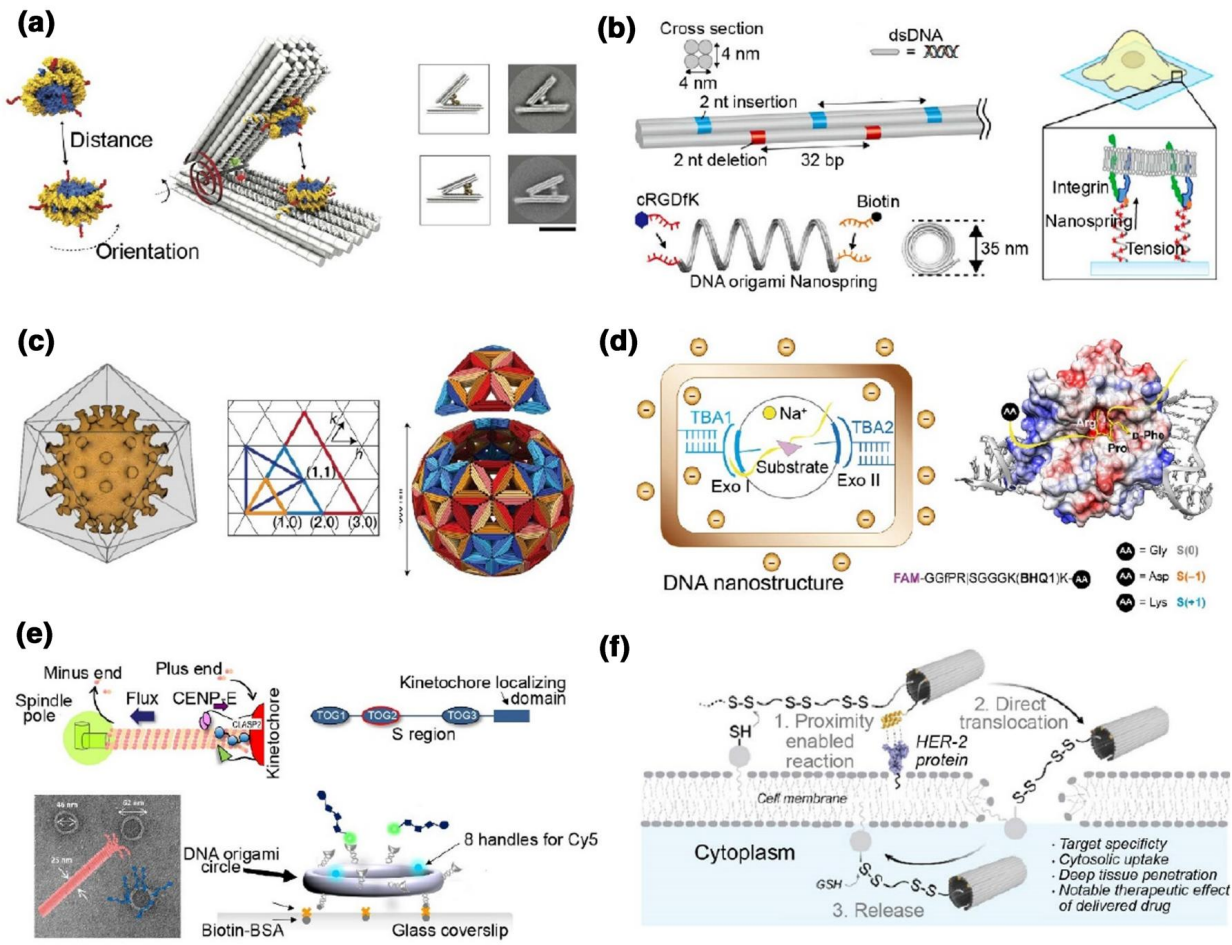
Artificial nanoprobes based on DNA origami that detect and modulate the specific biological behaviors. (a) A tweezer‐shaped DNA origami force spectrometer for studying nucleosome pair interactions^[21]^ (Copyright 2016, American Association for the Advancement of Science). (b) A coil‐like DNA origami sensor for the detection of the mechanical forces between cells and the substrates^[23]^ (Copyright 2023, American Chemical Society). (c) DNA origami shell for capturing entire viral particles and inhibiting their infection^[26]^ (Copyright 2021, Springer Nature). (d) Thrombin‐immobilized DNA origami structures for catalytic property study^[29]^ (Copyright 2022, American Association for the Advancement of Science). (e) DNA origami circles for investigation of microtubule dynamics^[30]^ (Copyright 2023, American Association for the Advancement of Science). (f) An affibody‐attached disulfide unit‐modified DNA origami device for enhanced cellular entering^[32]^ (Copyright 2023, American Chemical Society).

In 2016, Funke et al.^[21]^ created a tweezer‐like DNA origami scaffold for the integration of two nucleosomes, forming a nanoscale force spectrometer, to study nucleosome pair interactions and provide information for the impact on the genome regulation (Figure 2a). Kamińska et al.^[22]^ designed a strategy based on Förster‐type resonance energy transfer (FRET) pair‐integrated DNA origami probes for graphene energy transfer (GET) studies at the single‐molecule level. The authors constructed a pillar‐shaped DNA origami integrated with pyrene molecules for fixation of the whole origami probe on graphene, and with dye molecules attached at the pre‐designed sites to form the FRET pairs. Using the origami nanoprobes, the authors demonstrated GET tracking, enabling the isotropic precision down to the single‐molecule level. The combination of GET‐DNA PAINT technology for visualizing detailed structures with super‐resolution (∼2.5 nm of *z*‐direction provided by graphene quenching) was achieved. Matsubara et al.^[23]^ designed a coil‐like DNA origami sensor to study the mechanical forces between cells and the substrates. The authors constructed a rigid 4HB nanospring origami with a biotin‐labeled end to anchor the origami structure to an avidin‐coated surface. At the other end of the DNA nanospring, cRGD peptides were attached to interact with integrins expressed on human foreskin fibroblasts (HFFs). Live‐cell experiment was performed and mechanical properties of the origami at the cell‐substrate interface were studied. Using acoustic force spectroscopy (AFS), the authors estimated force‐extension curve of the origami and calculated the force over a broad range (from sub picoNewtons to 50 pN) (Figure 2b).

DNA origami‐based nanostructures can also be engineered for dynamic performance under specific internal/external signals, which have been used as nanoprobes for the detection of specific biochemical messengers. Liu et al.^[24]^ designed tweezer‐like DNA origami probes with gold nanorod (GNR) decorated on the arms, and specific DNA controlling moieties (containing disulfide bonds or aptamer sequences responsive to adenosine) were integrated into the origami‐based chiral probe, which was kept at the “locked” state until corresponding stimuli triggered the nanoprobes. In the presence of signal molecules (e.g., glutathione (GSH) or adenosine), the controlling components became “unlocked,” allowing the separation of the two GNR‐DNA arms of the DNA origami tweezers. The structural reconfiguration of the nanoprobes was converted to a plasmonic circular dichroism (CD) signal readout enabling the precise detection of the corresponding molecular inputs. The same group^[25]^ recently advanced their plasmonic origami nanoprobes by introducing a DNA logic circuit to recognize and amplify weak biochemical signals.

Besides small‐molecular triggers, DNA origami nanoprobes can be designed for recognitions of macromolecules. Sigl et al.^[26]^ designed a static virus‐like DNA icosahedral origami shell for capturing entire viral particles while inhibiting their infection of host cells (Figure 2c). In addition to those static origami shells, Engelen et al.^[27]^ provided a stimuli‐responsive strategy for the construction of reconfigurable DNA shells. The authors designed 20 identical triangular origami components, then assembled them via a shape‐complementary stacking approach to form a multi‐layered DNA origami shell and stabilized the shell structure by relatively high magnesium concentrations (25 mM MgCl_2_). These triangular components created by the authors revealed antigen pairs on their triangle‐triangle edges as bivalent binding sites, forming IgG antibody‐antigen connections. In the presence of soluble antigens, these IgG staples were replaced from the DNA shells, triggering the structural disassembly. As tools for biophysical studies, such as antibody‐antigen interactions, Zhang et al.^[28]^ constructed a type of 2D triangular antigen epitope‐displaying DNA origami assembly to study the transient interaction of the patterned epitopes with immunoglobulin Gs (IgGs). Using high‐speed atomic force microscopy (AFM) imaging, the authors found that the inter‐molecule distance within 3–20 nm was critical for the artificial epitope‐IgG interaction.

Dynamic DNA origami structures have been used to engineer nanoprobes and devices for studying enzymatic properties, protein assembly dynamics, particles' curvatures and cell entry. DNA origami nanostructures with designable inner pores or cavities can easily be applied as nanocontainers to precisely localize enzyme molecules and modulate their activities. Recently, Kosinski et al.^[29]^ chose thrombins as model molecules and created two types of DNA origami nanostructures, a 2D rectangle (65 × 90 nm) with internal space (26 × 27 nm), and a 3D box‐shaped structure (32 × 32 × 25 nm), to address and cage the enzymes via protruding thrombin‐binding aptamers. The authors found that the catalytic properties of encaged enzymes differed from those of their freely diffusing counterparts and were impacted by DNA/substrate electrostatic interactions, which were associated with the level of DNA‐protein tethering (Figure 2d). Luo et al.^[30]^ designed several DNA origami circles (∼46 nm or 62 nm in diameter) to form biological tools to study microtubule dynamics. The authors used human cytoplasmic linker‐associated proteins (human CLASPs) as model proteins, which can be precisely captured by DNA circles with pre‐designed handles. Using the origami immobilized on the glass coverslip, the kinetochore microtubule plus‐end and its coupling to the kinetochore were then studied. Their results showed that the clustering of CLASPs formed microtubules by total internal reflection fluorescence (TIRF) imaging (Figure 2e). The mechanical properties of these microtubules were also studied by laser trapping technology. A curvature‐sensing approach for determining particle size is provided by Büber et al.^[31]^ The authors designed a wing‐like DNA origami nanodevice with a flexible hinge connecting the two flat wing‐shaped blocks. Fluorescence resonance energy transfer (FRET) pair molecules, Cy3B and ATTO647 N, were precisely positioned at the inner edges of the wings. On the bottom side of the DNA nanodevices, functional components (including biotin molecules, cholesterol tags, or ssDNA) for particle attachment were precisely patterned. Using FRET technology as the signal transduction on the single‐molecule level, the authors adopted their origami devices as curvature sensors for particle size detection. In their results, different nanoparticles within a size range from 50 to 300 nm as well as the bending angle range from 50° to 180° could be determined by the origami sensors.

Recently, Yu et al.^[32]^ reported a DNA nanodevice that bypassed the endo/lysosomal trapping for direct cellular entry. They designed a 2D rectangular DNA origami (60 × 90 × 2 nm) as a nanocarrier with multiple protruding sites and desired structural curvature for cargo loading. At one end of rectangular origami carriers, human epidermal growth factor receptor 2 (HER2) affibody molecules (Kd = 22 pmol/L) were attached for enhancing the proximity and targeting specificity of the DNA structure to HER2‐positive breast cancer cells. Disulfide unit modification (a linear tert‐butyl disulfide with 6‐repeats, 6SS) was introduced to the ssDNA strand via phosphonamidite chemistry. The 6SS‐containing ssDNA sequences with designed overhangs were site‐specifically integrated into the extensions on the 2D origami carriers and shielded by the tubular structures driven by origami's curvature and disulfide units' hydrophobic interactions. Once origami nanocarriers brought disulfide units into close proximity to thiol groups on the cell surface, efficient disulfide exchange reaction and cytosolic uptake were induced. Their results showed rapid and direct internalization of HER2‐6SS‐DNA origami structures, enabling improved cellular delivery of shRNA (MCl‐1) and small molecular drugs (doxorubicin) (Figure 2f).

**FIGURE 3:**
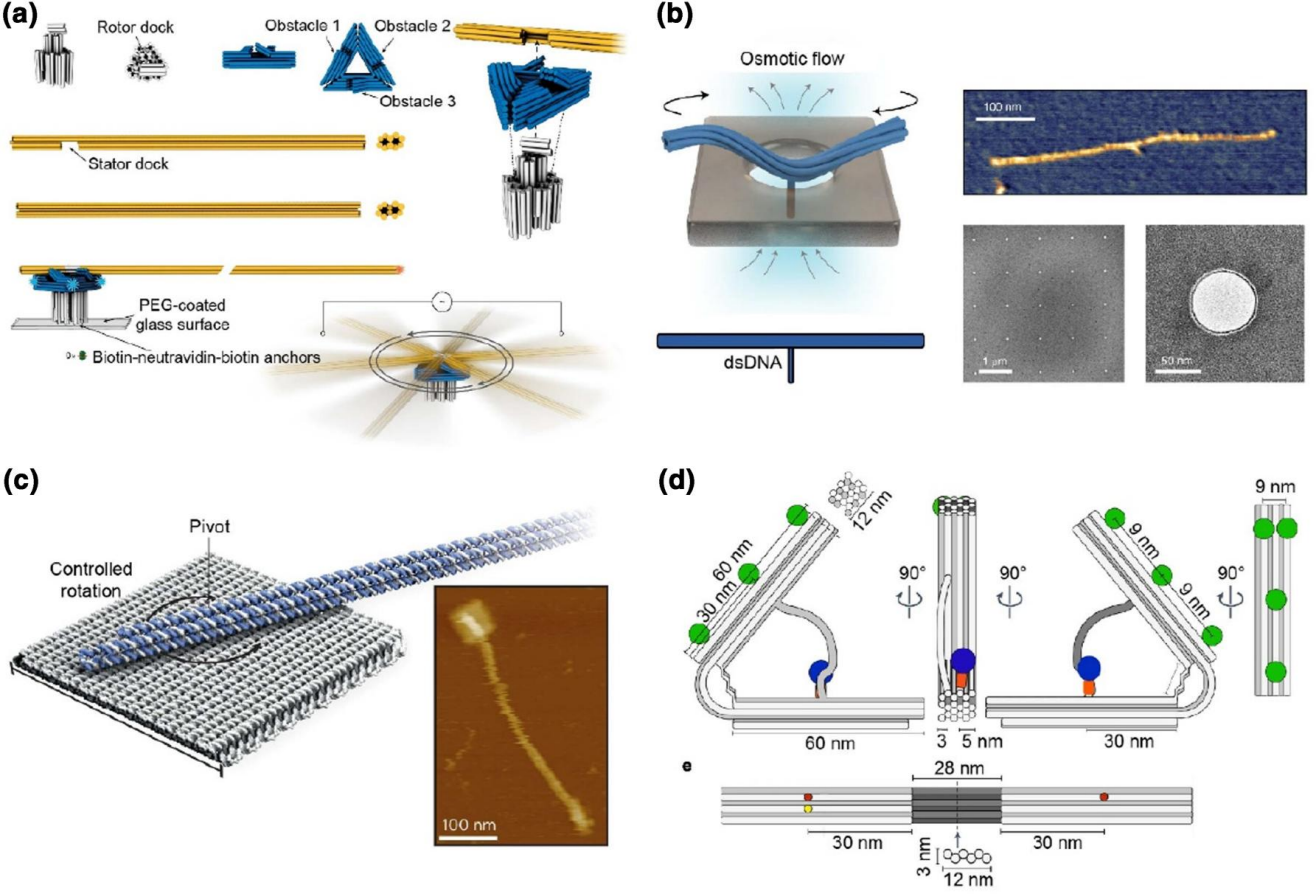
DNA origami nanodevices modulated in biomimetic fashions. (a) A ratchet‐like DNA origami as a nanoscale rotary motor^[33]^ (Copyright 2023, Springer Nature). (b) A T‐shaped solid‐state membrane docking origami for rotary motions^[34]^ (Copyright 2022, Springer Nature). (c) A long, rigid robotic origami arm that rotated in arbitrary directions^[36]^ (Copyright 2021, Springer Nature). (d) A tweezer‐like DNA engine driving a passive follower component^[38]^ (Copyright 2023, Springer Nature).

Another class of dynamic DNA origami nanostructures is inspired by many natural biological rotary motors in cells. Pumm et al.^[33]^ designed a ratchet‐shaped DNA origami to act as a nanoscale rotary motor. The authors designed a rigid multi‐layered DNA motor with three origami domains, a pedestal (40 × 30 nm), a triangular plate (60 × 13 nm) and a long, rigid, rod‐like rotary arm (∼550 nm). They fixed the pedestal origami domain onto a PEG‐coated glass surface through biotin‐neutravidin‐biotin anchors. At the other end of the pedestal, there was a protrusion through the central cavity of the triangular plate, displaying a docking site for the mechanical arm. Platinum electrodes were used to generate a square‐wave alternating current to power the origami motor (Figure 3a).

Shi et al.^[34]^ designed a T‐shaped DNA origami structure with a 6HB (450 nm‐long) and a dsDNA protrusion (50 nm‐long) at the central point of the T‐structure. The T‐shaped origami bundles were docked onto nanopores (50 nm‐diameter) in a thin solid‐state membrane. The authors demonstrated that nanoscale flow of water/ion through the nanopores can generate rotary motion of these T‐shaped origami bundles (Figure 3B). The same group then reported a similar strategy for studying rotary DNA nanostructures on nanopores. Inspired by natural rotary motors such as ATP synthases, Shi et al.^[35]^ constructed a DNA origami‐based transmembrane nanoturbine. The authors designed DNA origami‐based turbine structures, which can be docked on solid‐state nanopores and powered by a nanoscale hydrodynamic flow within the pores. These origami structures consisted of a central axle and 3 chiral blades (left‐ or right‐handed), with a height (24 or 27 nm) comparable to that of ATP synthase (∼20 nm). Their results suggested that a direct current (DC) voltage or a transmembrane ion gradient could be used as driving forces for the rotation of origami turbines, delivering tens of picoNewton nanometers of torque, which was similar to the natural rotary motors.

Vogt et al.^[36]^ presented an idea for the construction of rigid DNA nanomachines that enable rotational motions and are powered by external electric fields. Based on their previous work,^[37]^ the authors created 55 × 55 nm rectangular DNA origami plate structure to host a long, rigid DNA “arm” (∼463 nm in length), with the “arm” connected through a flexible joint positioned on the center of the DNA rectangle. The entire DNA robotic system was manipulated by an electric field, driving the rotary arm in arbitrary directions, which could be viewed by single molecular fluorescence microscopy. The authors investigated the mechanical features of DNA joints that acted as molecular torsion springs and demonstrated that the joints could store and release mechanical energies by winding up and relaxing motions (Figure 3c). Alternatively, Centola et al.^[38]^ designed a chemical input‐powered DNA origami‐based nanomachine that could drive a passive follower. The authors constructed a tweezer‐like DNA origami nanoengine, in which a DNA hinge connected with two rigid, tweezer arms (60 × 12 × 9 nm) formed by 18HB DNA. On the inner sides of the stiff arms, there were two separate binding sites, one for a T7 RNA polymerase (T7RNAP), and the other for a dsDNA template strand. The attached T7RNAP performed DNA‐templated RNA transcription locally on the DNA machine, consuming nucleoside triphosphates (NTPs) as fuel to produce energy for a rhythmic pulsating motion of two DNA origami arms. The actuation control of this DNA machine generated efficient force to transport the second origami component (Figure 3d).

DNA origami has been proven to be an elegant and powerful platform for the precise fabrication of versatile nanoprobes for biological signals and processes, including accurate nanosensors for mechanical force, specific biochemical messengers, and enzyme‐driven reactions in complicated biological environments. In addition, a series of dynamic nanodevices and nanomachines modulated in biomimetic manners have also been summarized. In particular, DNA origami structures offer many advantages over conventional materials. As unique addressable scaffolds, specific functional components (including aptamers, peptides, antibodies, etc.) can be integrated into the origami nanodevices to enhance the recognizing/binding properties of the nanodevices, and even amplify the efficacy for weak biochemical signals. Sophisticated nanostructures, such as origami tweezers, springs, circles, wings, cavities, tubes, etc., can be designed on‐demand, constructing exquisite origami geometries for corresponding mechanical modulation in biofluids or cellular environments. The dynamic design of the nanodevices with biological stimulus‐triggered reconfiguring features can be customized, enabling specific molecular detection or biomimetic motions.

## 3 DNA ORIGAMI NANOPORES FOR MIMICKING NATURAL MOLECULAR CHANNELS

**FIGURE 4:**
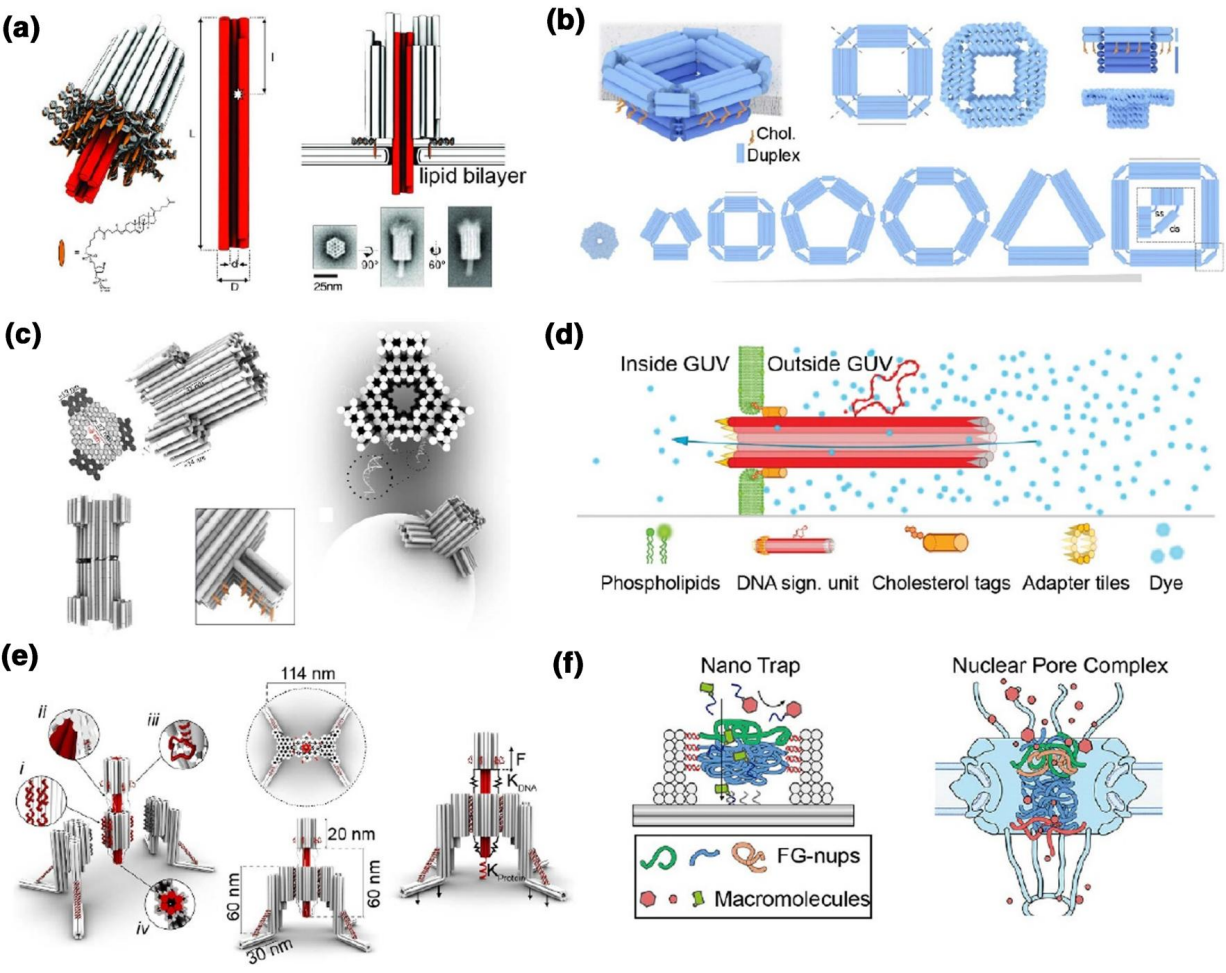
Bio‐inspired nanopores and selective barriers based on DNA origami. (a) A multi‐layered DNA origami nanopore constructed from a transmembrane origami channel and a membrane‐attaching cap^[40]^ (Copyright 2012, American Association for the Advancement of Science). (b) Several lipid‐inserted origami nanopores with tunable structures^[42]^ (Copyright 2022, Springer Nature). (c) An origami nanopore with DNA strand‐locked lipidated flaps^[43]^ (Copyright 2019, Springer Nature). (d) A μm‐long origami channel for cargo transportation^[47]^ (Copyright 2023, Wiley‐VCH). (e) A winch‐like, ligand‐decorated DNA origami nanodevice for investigation of ligand‐receptor interactions and cell signaling^[48]^ (Copyright 2022, Springer Nature). (f) An origami‐based, nuclear pore complex (NPC)‐like selective barrier^[50]^ (Copyright 2021, American Chemical Society).

Based on approximately 40 years of research into the structural design and creation of sophisticated molecular self‐assemblies, DNA nanotechnology is now being used to develop nanomaterials for further studies on the artificial membranes and biological membranes.^[39]^ In Section 2, we summarized recent advances in origami assemblies based on precise construction and dynamic modulation. These sophisticated nanostructures with elegant and pre‐designed geometries can also be utilized in mimicking natural molecular channels. DNA origami‐based techniques have been used to engineer nanoscale pores, gates and selective barriers that can act as artificial transport channels to control molecular exchanges. The first type of DNA origami‐based synthetic transmembrane channels was reported by Langecker et al.^[40]^ in 2012 (Figure 4a). The authors designed a multi‐layered DNA origami platform consisting of a stem‐shaped DNA origami with an inner cavity (∼2 nm of diameter) and a barrel‐like origami cap with cholesterol tags. These DNA nanostructures could be used to attach to lipid bilayer through their hydrophobic tags and control the passage of ions or single DNA molecules.

Based on the pioneering work, several advances have been made in the construction of bio‐inspired DNA nanopores and nanoholes with larger sizes, sensing properties and active motions. Lanphere et al.^[41]^ presented a detailed description of the design, construction and application of lipid‐engineering DNA origami‐based nanopores. Xing et al.^[42]^ constructed several structurally tunable nanopores for lipid membrane attachment. Their DNA nanopores contained two structural components: the outer‐membrane caps and the membrane‐spanning barrels. Those polygonal nanopores varied in shapes and sizes, with different lumen widths ranging from ∼6 to 20 nm. Cholesterol‐tagged DNA strands were anchored at the bottom surface of the cap components, guiding the insertion of nanopores into the lipid membrane. Using their DNA nanopores, direct single‐molecule sensing of proteins (with diameter of ∼10 nm) was achieved (Figure 4b).

A rigid DNA origami nanopore structure with an inner size of 9 nm was constructed by Thomsen et al.^[43]^ and this hexagonal origami lattice was designed with programmable lipidated flap components. In the closed state, the three DNA flaps on the surface of origami structures were locked by staple strands and shielded the hydrophobic moieties (the cholesterol tags) from the aqueous solution. Upon the addition of the corresponding‐opening ssDNA, the strand displacement was induced, and the flaps were extended, revealing the lipids to drive membrane insertion into small unilamellar vesicles (SUVs) and giant unilamellar vesicles (GUVs). Using optical technologies, the mechano‐sensitivity of DNA nanopores as well as the selective translocation of cargo molecules through DNA nanopores were studied (Figure 4c). Fragasso et al.^[44]^ designed and created circular DNA origami nanopores with a height of 10 nm and a wide inner diameter of 35 nm. After the incorporation of 32 sites of cholesterol molecules on the outer surface, these origami pores could be inserted into GUVs during their formation process by inverted‐emulsion techniques. These large membrane‐incorporated pores allowed macromolecules such as folded proteins to enter the GUVs. These pore‐containing liposomes showed optimal selectivity of cargo sizes, permitting molecules up to 28 nm in diameter to enter the GUV cavities and blocking the larger ones.

Dey et al.^[45]^ designed a programmable stimuli‐gated DNA origami channel to control the translocation of cargo molecules. The authors constructed a 70 × 70 nm single‐duplex layer rectangular structure with a mechanical DNA lid of 20.4 × 20.4 nm. Two DNA duplex locks were designed to keep the lid closed until the presence of key strands to open it. In the next step, the addition of the reverse key strands induced the strand displacement and closed the lid again. Cholesterol‐tagged ssDNA anchors were placed on the bottom side of the DNA squares for driving the lipid membrane insertion of SUVs and GUVs. Reversible gated properties, and protein transport through the DNA nanopores were studied using green fluorescence proteins (GFPs) as model molecules. Li et al.^[46]^ created micrometer‐scale nanochannels based on DNA origami technology for leakless transportation of cargo molecules. The authors designed DNA tubular structures with an inner diameter of 7 nm and a length of micrometers. With the hydrophobic modification at one end, the long DNA tubes can be spanned in the lipid bilayers, serving as transporting channels for nanofluids. Dye diffusion assays using optical characterization suggested that small molecular cargos could enter the GUV cavities from one end of the long DNA origami channels to the other. Jahnke et al.^[47]^ designed cholesterol‐tagged DNA origami tubes and inserted them into the phospholipid membranes of GUVs, forming nanopores. Their results showed that small molecular dyes could transfer into the GUVs through the transmembrane‐spanning origami tubes. After incorporation with biotins, these inserted origami tubes acted as mimic receptors, clustering upon the addition of streptavidin molecules. Next, the translocation of ssDNA strands was allowed from the outside of GUVs and the cargos filled into the GUV lumen. In response to chemical signals, the mechanical coupling and breaking of the artificial DNA cytoskeletons (DNA filaments) were triggered inside the GUVs (Figure 4d).

Besides DNA origami nanopores on the lipid bilayers, artificial channels and gates on the cell membrane or nuclear envelope have also been created and studied. Mills et al.^[48]^ constructed a winch‐like DNA‐based nanodevice. Three DNA origami domains were designed and then assembled a central cylinder origami with two landing legs in a 1:2 ratio. Cholesterol‐labeled ssDNA strands were placed at the bottom of the origami legs, adhering the fully assembled DNA nanoplatform to the lipid membranes of SUVs. These autonomous DNA nanowinches were applied to MCF 7 cells, a human breast cancer cell line, to study the ligand‐membrane receptor interactions and signaling. The authors modified cyclic RGD (cRGD) peptides on the central part of the DNA nanowinches, and then incubated the functional nanodevices with cells expressing integrins. The interaction of tagged cRGDs with integrins on the cell membrane triggered the phosphorylation of intracellular focal adhesion kinases (FAKs), which was detected by luminescence resonance energy transfer (LRET) techniques (Figure 4e). Liu et al.^[49]^ constructed several 400 nm‐long six‐helix bundle (6HB) DNA origami nanostructures, which were then connected end‐to‐end with each other to form micrometer‐long linear DNA structures. With cholesterol‐tagged ssDNA moieties, those prolonged DNA microtubes were attached to the membrane of pheochromocytoma (PC12) cells, thus facilitating artificial cell‐cell junctions based on DNA structures. The authors showed cell‐cell adhesion using confocal laser scanning microscopy (CLSM), then studied the exchange of small molecules and membrane vesicles through the cell conjunctions. They also detected chemical synaptic transmission between two connected PC12 cells by inserting carbon fiber nanoelectrodes (CFNEs) into the intercellular gaps. They found that the vesicular exocytosis was more active between the cell‐cell junctions than within a single cell.

Shen et al.^[50]^ constructed a nuclear pore complex (NPC)‐like DNA origami structure. Their nanotrap‐like origami consisted of two components, a square DNA channel with an inner diameter of 35 nm and a height of 17.5 nm, as well as a 57 × 55 × 5 nm DNA baseplate. The authors applied these structures to trap gold nanoparticles or phenylalanine‐glycine‐rich nucleoporins (FG‐nups) as biomimetic structures to study key properties of natural selective barriers (types, densities, spatial patterns of multiple motifs, etc.) (Figure 4F). Recently, the same research group^[51]^ reported a further design of NPC‐like origami nanochannels for studying the mechanisms by which viruses such as HIV‐1 enter the cell nucleus. The authors constructed three types of DNA origami channels, a ring‐shaped cylinder (with an inner diameter of 45 nm and a height of 14 nm), and two open‐ended octagonal prisms (one with an inner diameter of 40 nm, the other with an inner diameter of 60 nm, and the two channels with a height of 30 nm). The ssDNA handles for capturing and immobilizing of nucleoporins (nups) were precisely designed and positioned on the inner surfaces of the DNA origami channels that served as the scaffolds for NPC mimics. The authors used DNA origami to guild several ssDNA (termed as anti‐handle)‐tagged nups (including Nup358, Nup62 and Nup153) for site‐specific assembly to the inner cavities of the origami channels. Their systems were used to study the interaction (including docking, attachment, insertion, etc.) of viral capsids to the artificial NPCs modified with multiple nups.

These abovementioned DNA origami‐based nanopores, channels, holes and barriers have been designed, constructed and used to control molecular exchanges as active participants in biomimetic events and processes. In the future, DNA nanotechnology will provide more smart materials for further elegant modulation of biological signaling and reactions, including biosensing and on‐demand drug release.

## 4 DNA ORIGAMI‐GUIDED BIOMOLECULAR PATTERNS FOR REGULATION OF BIOLOGICAL FUNCTIONS

In living systems, biological information transfer processes extensively rely on interaction between soluble ligands and cell membrane‐bound receptors.^[52]^ The precise spatial control of ligand molecules is considered significant for cellular signaling, which may trigger the downstream intracellular regulatory or therapeutic functions.^[53]^ In Sections 2 and 3, elegant DNA origami nanodevices and bio‐inspired nanopores have been summarized and discussed. In addition to the programmed and uniform geometries, DNA origami technology can provide a pre‐designed “canvas” to anchor desired components with nanoscale precision. Taking the unique addressability of DNA origami nanostructures, several types of origami‐based systems have been constructed for the precise anchoring of ligands and facilitating specific functions.[^10^, ^12^, ^13^, ^14^] DNA origami scaffolds, as nanoscale drawing boards for site‐specific arrangement of model ligands (usually via hybridization at pre‐designed locations), have been applied to study the intracellular signaling triggered by the engineered extracellular ligand‐origami constructs.

**FIGURE 5:**
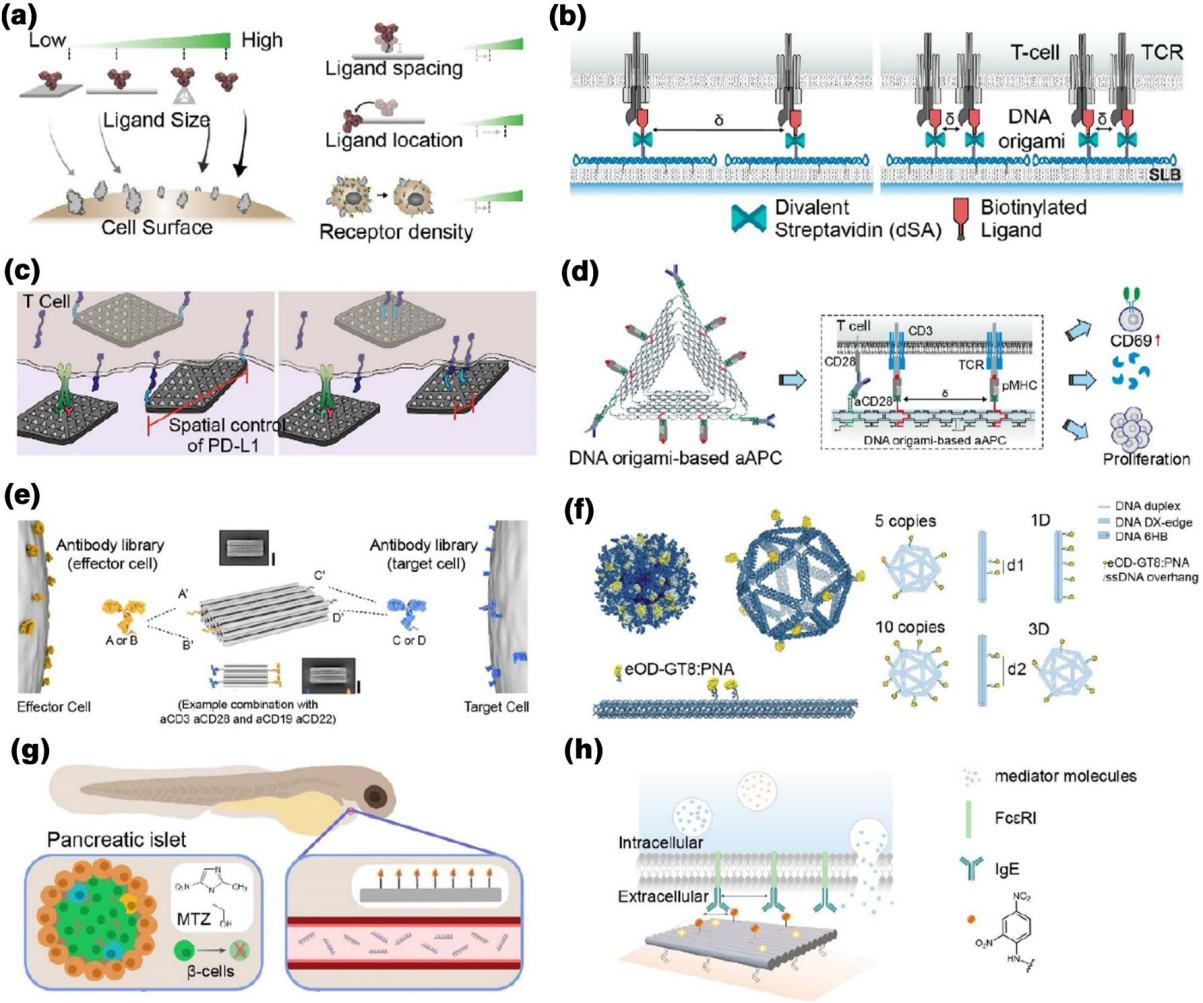
DNA origami nanostructures as addressable templates for guiding functional assemblies. (a) Various antibody‐attached DNA nanoassemblies for studying their interaction with receptors on cell membranes^[54]^ (Copyright 2021, American Chemical Society). (b) Precise organization of pMHC ligands on DNA origami for efficient TCR triggering^[55]^ (Copyright 2020, National Academy of Sciences of the United States of America). (c) PD‐L1 ligand‐displaying DNA origami for study T‐cell signaling^[56]^ (Copyright 2021, American Chemical Society). (d) Co‐assembly of pMHC ligands and aCD28s on DNA origami for construction of aAPCs^[57]^ (Copyright 2022, American Association for the Advancement of Science). (e) Multiple antibodies co‐assembly on DNA origami for a T‐cell engager^[60]^ (Copyright 2023, Springer Nature). (f) Precise patterned antigens on DNA origami for BCR activation^[61]^ (Copyright 2020, Springer Nature) (Copyright 2023, Springer Nature). (g) Insulin peptide‐decorated DNA origami for IR pathway activation and transcriptional response modulation^[62]^ (Copyright 2023, Springer Nature). (h) IgE antigen‐localized DNA origami for multivalent receptor binding and clustering on the mast cells^[63]^ (Copyright 2023, American Chemical Society).

Cremers et al.^[54]^ developed a site‐specific strategy to guide the assembly of a variety of model ligands (e.g., multiple antibodies) and investigated ligand‐spacing parameters affecting their interaction with the membrane receptors (e.g., programmed cell death protein 1 (PD1), epidermal growth factor receptor (EGFR), and human epidermal growth factor receptor 2 (HER2)). The authors fabricated multiple DNA nanostructures, including DNA nanorods, tetrahedrons, and rectangles, which were used as the ligand‐assembled templates, on which the pre‐designed DNA handle strands were extended to organize the ssDNA attached antibodies (Figure 5a). In contrast to soluble antibodies, their results showed that the antibodies assembled on the DNA nano‐templates lowered the absolute number of cell membrane‐bound receptors, which was due to that DNA nanostructures affect the accessibility of ligands to the bound receptors. Subsequently, the authors demonstrated that defined ligand‐spacing, which was controlled by the ssDNA patterns, was the critical parameter for ligand‐receptor interaction.

Hellmeier et al.^[55]^ demonstrated that DNA origami technology enabled the precise spatial organization of nanoarchitectures of peptide‐major histocompatibility complex (pMHC) ligands. In this study, the authors identified that the precise ligand arrangements were significant for effective triggering of T cell receptors (TCRs). Using pMHC‐origami nanostructures, efficient T‐cell activation was observed with the smallest unit consisted of two ligated TCRs within 20 nm (Figure 5b). Using DNA origami‐based wireframed rectangles as designed templates, Fang et al.^[56]^ provided another example to facilitate the spatial control of ligands, for example, programmed death‐ligand 1 (PD‐L1) molecules and study their effects on T‐cell signaling. In their study, CD3 and CD28 antibody‐displaying DNA origami was assembled to trigger efficient T‐cell signaling and activation. The authors found that a single PD‐L1 protein displaying on DNA origami or two ligands with an inter‐distance of 13 nm or 40 nm could not suppress the T‐cell signaling induced by CD3/CD28 antibodies, whereas co‐treatment with the PD‐L1‐origami with ligand spacing of ∼200 nm inhibited the T‐cell signaling. The inter‐ligand distance of those PD‐L1‐origami structures also affected the formation of PD‐1 nanoclusters and subsequent PD‐1/PD‐L1 signaling (Figure 5c).

Several different types of ligand‐integrated DNA origami structures have been designed for the modulation of immune responses. Sun et al.^[57]^ constructed artificial antigen‐presenting cells (aAPCs) for adoptive T cell therapy, based on DNA origami technology by co‐assembling pMHC ligands and an anti‐CD28 antibodies (aCD28) on 2D triangular DNA origami templates. The pMHC components (the TCR ligands) were attached at the three edges of the triangular DNA templates, whereas the aCD28 molecules (the co‐stimulatory ligands) were anchored at the vertices of the origami scaffolds, forming the origami‐based aAPCs. The effects of ligand organization via the origami addressability on T‐cell activation, including the copy number of ligands and the inter‐ligand spacing, were studied. The authors found that a shorter inter‐ligand distance could induce a stronger T‐cell response (Figure 5d). They also used their origami aAPCs for antitumor evaluation both in vitro and in vivo, eliciting efficient therapeutic effects. Another study^[58]^ by the same group introduced a similar origami‐based pMHC multimer as a reagent for antigen‐specific T‐cell detection. The authors created 2D triangular origami scaffolds to load pMHC molecules precisely controlling their valency and the inter‐molecule distance on the nanoscale addressable templates. Their results showed that the interaction of pMHC multimers with TCRs was enhanced once increase the pMHC copies and decrease the inter‐ligand spacing. Their origami‐based pMHC multimers were used in mouse models and T cells expressing low‐affinity TCRs were able to be determined. Besides controlling the ligands, fine‐tuned co‐receptor molecules with nanoscale precision can be achieved and have been used in studying antigen recognition.^[59]^ Using purified protein systems and cellular systems, Rushdi et al. demonstrated that the cooperative binding function of CD4 to TCR‐pMHC interaction. By controlling molecular spacing of TCR and CD4, they found a 7‐nm inter‐molecule distance optimized the formation of TCR‐pMHC‐CD4 bond, which was consistent with the tri‐molecular crystal structures.

Wagenbauer et al.^[60]^ constructed a T‐cell engager by programmable assembly of IgG/F(ab)/scFv antibodies with precise spatial arrangement. The authors provided a library of DNA‐conjugated proteins for site‐defined assembly antibodies on DNA origami nanostructures. They selected several antibodies (e.g., anti‐CD3, anti‐CD28 and anti‐CD137) for binding and activating effector T cells. For recognizing and targeting distinct target cell lines, another set of antibodies (such as anti‐CD19, anti‐CLL1, anti‐CD22 and anti‐CD123) were utilized. After antibody modification and origami‐antibody construct screening in vitro, the capacity of T‐cell engagers was determined by crosslinking the T cells and the target cells, subsequently resulting in efficient T‐cell mediated lysis to the target cells. At the animal level, these origami‐based engagers elicited T‐cell activation and controlled tumor outgrowth (Figure 5e).

Besides the abovementioned nanoassemblies for T‐cell‐based immune modulation, other types of macromolecules such as antigens for B‐cell signaling and functional peptides have been engineered by addressable DNA origami for ligand‐receptor signaling studies. Veneziano et al.^[61]^ designed several DNA origami‐based nanostructures to investigate immunoglobulin‐M (IgM)‐B‐cell receptor (BCR) activation. The authors constructed two types of origami particles, 1D DNA rods (∼80 nm in length) and 3D icosahedral DNA frameworks (∼40 nm in size) to site‐specifically organize the ssDNA attached‐model antigen molecules (an engineered outer domain of the HIV‐1 glycoprotein‐120, named eOD‐GT8). Using these origamus platforms, the authors investigated the copy numbers, inter‐molecule spacing of antigens and their spatial presentation on the addressable origami templates as well as the geometry/rigidity of DNA origami templates, the key parameters that significantly affect B‐cell signaling. They demonstrated that displaying only 5 copies of eOD‐GT8 antigens on 40 nm size viral‐shaped origami scaffolds maximized the B‐cell activation. Additionally, inter‐antigen distance on the origami scaffolds and their rigidity was also critical for BCR activation. 25–30 nm of antigen spacing and rigid DNA template for antigen presentation elicited robust B‐cell responses (Figure 5f).

For the investigation of insulin receptor activation, Spratt et al.^[62]^ constructed DNA origami nanostructures decorated with multiple insulin peptides, which are the ligands of insulin receptors (IRs) and are essential for lipogenesis, glycogenesis and other anabolic, biosynthetic processes. The authors designed a rod‐shaped origami scaffold (∼140 nm in length) as a guiding template for the precise organization of the insulin molecules with ssDNA tags. They used DNA point accumulation in nanoscale topography (DNA‐PAINT) technology to image the insulin molecules attached to the DNA origami rods and surface plasmon resonance (SPR) to study the interaction of insulin‐origami with IRs. They found insulin valency and spatial organization controlled by the origami scaffolds influenced the IR pathway activation and modulated the transcriptional responses. Furthermore, with the use of these origami‐integrated insulin multimers in β‐cell‐ablated zebrafish larvae, the authors demonstrated that their treatment elicited a reduction in glucose level in vivo (Figure 5g). Wang et al.^[13a]^ developed a DNA origami platform decorated with precisely patterned peptide ligands for modulating the fate of tumor cells. The authors designed two types of origami flat sheets (a single‐layer wireframe and a double‐layer square lattice) that displayed ssDNA handles for the assembly of complementary ssDNA‐conjugated ligands. Tumor necrosis factor (TNF)‐related apoptosis‐inducing ligand‐mimicking peptides were chosen as model molecules, which were site‐specifically located on the origami templates via hybridization with the ssDNA‐tagged ligands. Similar to those immune‐modulation systems, the predesigned ligand nanopatterns using the DNA origami templates were essential for their biological functions. Using human breast cancer cell lines (MCF‐7, SK‐BR‐3 and MDA‐MB‐231), it was demonstrated that peptides with a hexagonal pattern and an inter‐molecule spacing approximately 5 nm on the origami scaffold could trigger the death receptor clustering on the cell membrane, and thus induce apoptosis.

Schneider et al.^[63]^ developed a rectangular DNA origami template to precisely localize antigens to study their multivalent binding with immunoglobulin E (IgE) antibody molecules bound on mast cell membrane. The clustering of FcεRIs, IgEs' transmembrane receptors, could be mediated by multivalent binding of their artificial antigen‐decorated structures through specific interaction of origami‐bound antigens with antigen‐binding fragments (Fab) of FcεRI‐bound IgE antibodies. Similar to those abovementioned studies, the authors also used DNA origami techniques to facilitate the valency and spatial control of these nanoscale multiple ligand‐decorated architectures. They found that a molecular spacing of ∼16 nm between haptens, the artificial ligands, induced the stable interaction detected by SPR analysis. Furthermore, in cellular models (rat basophilic leukemia cells), it was observed that the binding of origami‐based ligand multimers was not dependent on the inter‐molecular spacing, indicating a supramolecular oligo‐valent nature of this interaction (Figure 5h). These origami‐based assemblies described above provide researchers with a series of intelligent tools to study biochemical processes and signaling on the biological interfaces.

## 5 OUTLOOK

After decades of research on the design and fabrication of sophisticated molecular self‐assembled structures, DNA origami has been proven to be an incredible technology for building smart materials with programmable functionalities and fascinating applications.^[8d,8e]^ DNA origami architectures, featured with outstanding structural programmability and versatility, have been exploited as nanoscale pegboards for precise arrangement of functional moieties, offering enormous opportunities for the creation of novel bio‐inspired materials. In this minireview, we have summarized the recent intriguing progress in precise construction of DNA origami‐guided materials for functional regulation on the biological interfaces, including DNA nanoprobes and nanodevices for precise biosensing and desired structural motions, DNA nanopores and gates for selective delivery of molecules through artificial or biological membranes, as well as DNA origami‐based arbitrary molecular patterns for biophysical/biochemical studies on the cell interface.

Taking advantage of their intrinsic programmability, a number of sophisticated DNA origami structures with varied sizes and shapes have been designed and fabricated to realize desired structural motions and biosensing applications,[^22^, ^23^, ^31^, ^33^, ^34^, ^36^] as well as selective control of the transmembrane molecule exchanges.[^35^, ^41^, ^42^, ^43^, ^45^, ^46^, ^47^] Through in silico design, origami‐based nanopores and channels with different topological features can be made to allow the access of molecules in the appropriate range of sizes. After the integration of stimulus‐responsive motifs, the DNA origami platforms have also been endowed with the ability to dynamically change and mimic biological processes. These studies suggest that DNA origami‐based materials have the potential to be the rationally designed platforms for studying the transmembrane substance transportation and simulating microscopic biological activities.

Special attention should be paid to the unique advantage of DNA origami as a displaying platform for presenting multiple functional components with pre‐designed valency, multimerization, inter‐molecule spacing and position. In addition to the thoroughly discussed biomimicking interactions involving T‐cell[^55^, ^60^] and B‐cell activation,^[61]^ the origami‐guided biomolecular assemblies have been employed in other ligand‐receptor binding related biological activities such as innate immune activation and coagulation. The oligodeoxynucleotides containing cytosine‐phosphate‐guanosine motif (CpG motif) is a ligand that triggers the activation of endosomal Toll‐like receptor 9 (TLR9), a sort of pattern recognition receptor. In a recent report, Comberlato et al.^[64]^ created DNA origami nanoparticles that present CpG motifs at an optimized inter‐molecule distance of 7 nm to achieve stronger activation of TLR9 in RAW 264.7 macrophages. Coagulation is one of the major challenges for safe and effective hemodialysis treatment. Zhao et al.^[65]^ described a DNA origami‐based aptamer nanoarray that enabled the inhibition of thrombin activity and thrombus formation. Two types of thrombin‐binding aptamers, recognizing the two exosites of thrombin molecules, were decorated onto origami structures with optimized distances and numbers, exhibiting enhanced anti‐coagulation in human plasma, fresh whole blood and a murine model.

Despite numerous optimal advances, there are remaining challenges and questions that need to be further answered in this exciting field. For instance, the structural stability and integrity of DNA origami scaffolds in complicated biological fluids still need to be studied and enhanced, especially when they are readily working in vitro and in vivo. Several reported approaches are based on chemical modification of structural DNA strands that fold for DNA nanoarchitectures, and these methodologies including phosphorothioate linkages^[66]^ and 2′O‐methyl ribose modifications^[67]^ have been used in clinically approved nucleic acid drugs. Alternatively, PEGylated polymer‐absorbed approaches have been introduced to improve the stability of DNA origami, and these polymer‐covered DNA nanostructures were reported with enhanced enzymatic resistance and prolonged circulating half‐life in vivo.^[68]^ Photo‐cross‐linking of the DNA nanostructures that contain acrydite modification^[69]^ or thymines at nicks in close proximity^[70]^ could be also employed for stability improvement.

In addition, despite recent careful investigations, the in vivo pharmacokinetics, biodistribution, metabolism and clearance mechanisms of DNA origami nanostructures are not clearly elucidated, which are crucial for further biological and biomedical applications. The precise interactions of DNA origami nanostructures with macromolecules in biochemical environments need to be intensively investigated. Although chemical synthesis and biological fermentation approaches have been reported,^[71]^ the cost of DNA production and structural fabrication is still higher than that of conventional nanomaterials including lipid nanoparticles (LNPs) and polymers, etc. Compared to those materials, the large‐scale good manufacturing practice (GMP)‐compliant production of DNA origami nanomaterials has not yet been achieved. From the laboratory to industrial scale, there is still a need for robust, reliable, and reproducible approaches for the mass production of DNA raw materials and functional nanostructures at low cost.

## CONFLICT OF INTEREST STATEMENT

The authors declare no conflicts of interest.

## Data Availability

Data sharing is not applicable to this article as no new data were created or analyzed in this study.
